# Diabetes associated with HPV infection in women aged over 50 years: A cross-sectional study from China’s largest academic woman’s hospital

**DOI:** 10.3389/fendo.2022.972963

**Published:** 2022-11-14

**Authors:** Chaoyan Yue, Chunyi Zhang, Chunmei Ying, Hua Jiang

**Affiliations:** ^1^ Department of Laboratory Medicine, Obstetrics and Gynecology Hospital of Fudan University, Shanghai, China; ^2^ Department of Gynecology, Obstetrics and Gynecology Hospital of Fudan University, Shanghai, China

**Keywords:** diabetes, prediabetes, HPV, HbA1c, vaginitis

## Abstract

**Background:**

Metabolic disturbances and immune alterations caused by diabetes are not just bystanders of HPV infection, but the conclusion that diabetes increases the risk of HPV infection requires more clinical epidemiological evidence to confirm. Our aim was to evaluate the association of diabetes with HPV infection risk in female patients aged over 50 years in the cervical clinic.

**Methods:**

We conducted a cross-sectional study of 6402 women aged over 50 years in the cervical clinic between May 2019 and March 2022 from China’s largest academic woman’s hospital. The quantitative-effect relationship between diabetes and HPV infection was observed by dose-response graph. Segmented multivariate logistic regression analysis was conducted to estimate the relative risk of HPV infection in diabetes patients. Multivariable predicted marginal proportions from logistic regression models were used to compute adjusted risk ratios.

**Results:**

There is a nonlinear relationship between HbA1c and the risk of HPV infection. When the HbA1c exceeds 5.7%, there is a saturation effect. After adjustment for confounders, the risk ratio for HPV infection in women with prediabetes was 1.09 (95% CI: 1.00-1.18) compared with women with HbA1c <5.7%, and the risk ratio for HPV infection in women with diabetes was 1.18 (95%). CI: 1.04-1.33). Sensitivity analysis showed that the risk ratio for HPV infection was 1.47 (95% CL: 1.07-1.91) when diabetes was associated with vaginitis. E-value analysis suggested robustness to unmeasured confounding.

**Conclusions:**

Diabetes and prediabetes are at increased risk of coinfection with HPV in female patients aged over 50 years in the cervical clinic.

## Introduction

The prevalence of diabetes has increased dramatically globally, affecting more than 8% of the world’s adults (1), and in China 2018 data suggest that the prevalence of diabetes has risen to 12.4%, the prevalence of prediabetes has reached 38.1%, the combined prevalence of diabetes and prediabetes is 50.5%, the median age is 51.3 years, and the weighted percentage of women is 49.5% (2). The aging of the Chinese population is the main reason for the prevalence of diabetes in China, and the prevalence of diabetes increases with age (3–6). Hemoglobin A1c (HbA1c) is the current gold standard for monitoring glycemic control and is now recommended for diagnosing diabetes and identifying individuals at risk for diabetes (7). HbA1c reflects the average blood glucose level over the past 2-3 months. Compared to fasting blood glucose (FPG), HbA1 c testing is convenient (no fasting required), has minimal daily variability, higher pre-analytical stability, and fewer daily interferences during stress, diet, or illness. No rapid or timed sampling is required; measurements are standardized, and correlate more closely with chronic complications (8).

Diabetes can lead to metabolic disorders and immune alterations that may be potential triggers for persistent HPV infection, diabetes rather than it being just a “bystander” of the infection of HPV (1). However, the conclusion that diabetes promotes HPV infection lacks clinical epidemiological evidence, and there are few reports on the potential association between diabetes and HPV, and the results are controversial.

To elucidate the risk of persistent HPV infection in women with diabetes or prediabetes, our cross-sectional study, using HbA1c as a diagnostic criterion for diabetes, explored the association between diabetes and HPV in female cervical outpatients over 50 years of age, and risk ratios adjusted for confounders were calculated by multivariate predictive marginal proportions from a logistic regression model.

## Methods

### Design and participants

Our study was a cross-sectional study with a total of 6402 women aged over 50 years between May 2019 and March 2022 at the Obstetrics & Gynecology Hospital of Fudan University (Shanghai, China), which was China’s largest academic woman’s hospital. Inclusion criteria: HPV-DNA examination and HbA1c determination were performed at our cervical clinic, aged over 50 years. Study exclusion criteria were as follows: HPV and HbA1c related records were not performed. Clinical data and results were obtained from Hospital Information System (HIS) and Laboratory Information Management System (LIS). All patients in this study were anonymous, in accordance with the principles of the Declaration of Helsinki and approved by Ethics Committee of the Obstetrics & Gynecology Hospital of Fudan University.

### Variables and measurements

HbA1c results were used as exposure factors in this study. HbA1c was analyzed using the Hemoglobin Testing System (VARIANT II, Bio-Rad). Confounding factors included age, BMI, history of adverse pregnancy, vaginitis, and syphilis. According to the American Diabetes Association, an HbA1c level of 6.5% or higher defines diabetes. An HbA1c level of 5.7%-6.4% was defined as prediabetes.

### Outcomes and measurements

HPV testing was performed in the pathology department with 1 of 2 HR-HPV testing methods during the study period: the Cobas 4800 system (Roche Diagnostics), or BioPerfect (Bioperfectus Technology). Any type of HPV positive is considered a positive event.

### Statistical analysis

Data are presented as means (SD) for continuous variables and percentages (%) for dichotomous variables. A smooth curve fit plot of HbA1c was created to examine the shape of the relationship between HbA1c and HPV. We applied a two-segment linear regression model to test the saturation effect of HbA1c on HPV based on smoothing plots. A segmented regression model was then used to compare the differences between models I and II by performing log-likelihood ratio tests for the single-linear linear regression model and the two-segment linear model. P-value<0.05 means Model II is significantly different from Model I, which indicates a non-linear relationship. We used multivariable predicted marginal proportions from logistic regression models to compute adjusted risk ratios for the association between HbA1c and HPV. The models were adjusted for age, obesity, abnormal pregnancy history, vaginitis, history of syphilis. The robustness of these findings was assessed in multiple sensitivity analyses. First, Subgroup analyses were performed according to BMI and vaginitis as stratification factors, and interaction tests assessed whether the relationship between diabetes and HPV was consistent across subgroups. Second, we explored the potential for unmeasured confounding between diabetes and HPV by calculating E-values (9). The E-value quantifies the required magnitude of an unmeasured confounder that could negate the observed association between diabetes and HPV. P-values <0.05 were considered statistically significant. All reported P-values are bidirectional. Software IBM SPSS (version 21.0. IBM; Armonk, NY) and Stata (version 14.2, StataCorp LP, College Station, TX, USA) were used for statistical analysis.

## Results

Our study included 6402 cervical outpatients over the age of 50, with a mean age of 58.01 ± 6.51 and an HPV-positive rate of 34.16% (2187 cases). Mean glycated hemoglobin was 5.79 ± 0.75%. **Table 1** describes the baseline characteristics of the subjects, and some clinical characteristics that may be associated with the occurrence of HPV, such as BMI, adverse maternal history, vaginitis, and history of syphilis.

**Table 1:** Baseline characteristics of the study participants.

	HbA1c<5.7	5.7≤HbA1c<6.5	HbA1c≥6.5	*p*-value
No. of participants	3135(48.97%)	2651(41.41%)	616(9.62%)	
Age	56.65 ± 6.10	58.90 ± 6.44	61.10 ± 7.04	<0.001
HBA1C	5.33 ± 0.26	5.94 ± 0.20	7.48 ± 1.15	<0.001
HPV				0.16
Negative	2096 (66.86%)	1729 (65.22%)	390 (63.31%)	
Positive	1039 (33.14%)	922 (34.78%)	226 (36.69%)	
Obesity				<0.001
No	3109 (99.17%)	2615 (98.64%)	599 (97.24%)	
Yes	26 (0.83%)	36 (1.36%)	17 (2.76%)	
Abnormal Pregnancy History				0.48
No	2117 (67.53%)	1808 (68.20%)	431 (69.97%)	
Yes	1018 (32.47%)	843 (31.80%)	185 (30.03%)	
Vaginitis				0.52
Negative	2980 (95.06%)	2520 (95.06%)	579 (93.99%)	
Positive	155 (4.94%)	131 (4.94%)	37 (6.01%)	
History of syphilis				0.14
Negative	2393 (98.60%)	1998 (97.99%)	472 (97.52%)	
Positive	34 (1.40%)	41 (2.01%)	12 (2.48%)	

**Figure 1:**
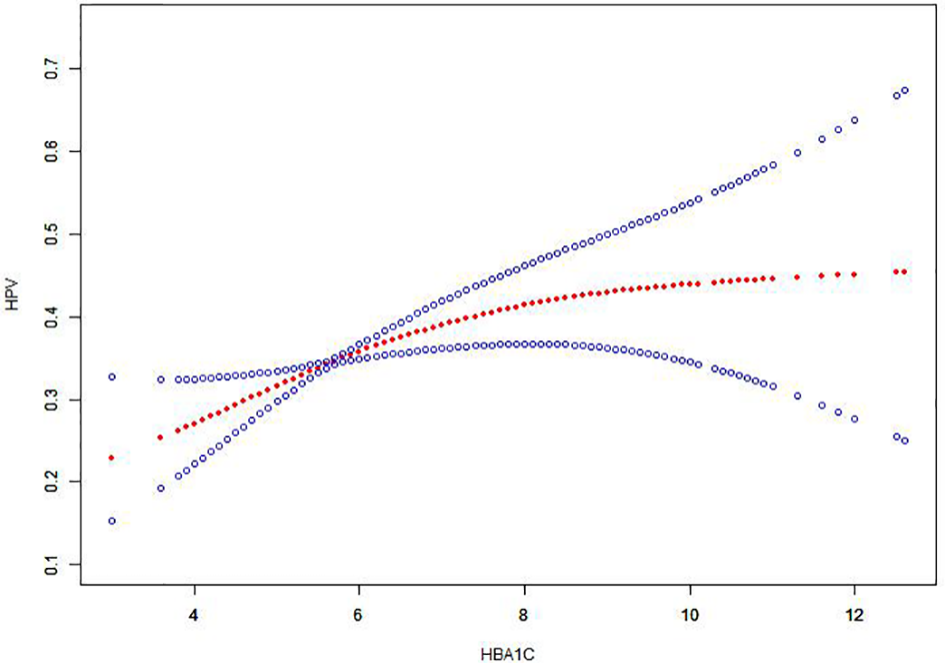
The association between HbA1c and HPV. Note: The red line represents the fitted curve of HbA1c and HPV, and the blue line represents the 95% confidence interval of the curve.

A piecewise regression model between HbA1c and HPV found a saturation effect when HbA1c was 5.7%. When HbA1c<5.7%, for every 1% increase in HbA1c, the risk of HPV positivity increased by 44% (OR=1.44, 95%CL: 1.12, 1.84). When HbA1c≥5.7%, for every 1% increase in HbA1c, the risk of HPV positivity increased, the risk was increased by 7% (OR=1.07, 95%CL: 0.97, 1.18). Log-likelihood ratio test: P<0.001, there is a non-linear relationship between HbA1c and HPV risk (**Table 2**). The dose-response relationship between HbA1c and HPV-positive risk was shown by smoothed splines (**Figure 1**). The relationship between HbA1c and HPV positive risk was S-type. When HbA1c ≥ 5.7%, the change in HPV positivity rate becomes moderate

**Table 2:** Threshold effect analysis for the relationship between HbA1c and HPV.

Models	Risk of HPVAdjusted OR (95%CI)	*p-*value
Model I		
One line slope	1.13 (1.05, 1.23)	0.002
Model II		
Turning point	5.7	
< 6 slope 1	1.44 (1.12, 1.84)	0.004
> 6 slope 2	1.07 (0.97, 1.18)	0.14
Slope 2 – Slope 1	0.75 (0.56, 0.99)	0.046
Predicted at 6	-0.59 (-0.67, -0.51)	
LRT test	0.044	

Model I, linear analysis; Model II, non-linear analysis. LRT test: Logarithmic likelihood ratio test. (p-value<0.05 means Model II is significantly different from Model I, which indicates a non-linear relationship); adjust for age, BMI, abnormal pregnancy history, vaginitis, history of syphilis.

After adjustment for confounders, the risk ratio for HPV infection in women with prediabetes was 1.09 (95% CI: 1.00-1.18) compared with women with HbA1c <5.7%, and the risk ratio for HPV infection in women with diabetes was 1.18 (95%CI: 1.04-1.33) (**Table 3**).

**Table 3:** Risk Ratios for HPV among different level of HbA1c.

Exposure	N	No. of Patients with HPV (Event Rate)	Risk Ratio (95% CI)	*p*	Ajust Risk Ratio (95% CI)	*p*
Continuous HbA1c	6402	2187 (34.16%)	1.06 (1.02- 1.10)	0.003	1.08 (1.04-1.14)	0.001
Clinical cutoffs						
HbA1c <5.7	3135	1039 (33.14%)	Reference		Reference	
5.7≤HbA1c<6.5	2651	922 (34.78%)	1.05 (0.98-1.12)	0.098	1.09 (1.00-1.18)	0.03
HbA1c≥6.5	616	226 (36.69%)	1.11 (0.98-1.23)	0.048	1.18 (1.04-1.33)	0.006

Ajusted for age, BMI, abnormal pregnancy history, vaginitis and history of syphilis.

Sensitivity analyses by BMI and vaginitis found that the effect of HbA1c on HPV was consistent between subgroups (**Table 4**). There was no interaction between the different subgroups (BMI: p=0.63 for the interaction; vaginitis: p=0.22 for the interaction). We generated an E-value to assess the sensitivity to unmeasured confounding. The primary findings were robust, unless an unmeasured confounder existed with an RR greater than 1.68.

**Table 4:** Subgroup analysis of Risk Ratios for HPV.

HPV	n	No. of Patients with HPV	Risk Ratio (95% CI)	*p*	*p* for interaction	Ajust Risk Ratio (95% CI)	*p*	*p* for interaction
Obesity					0.79			0.63
BMI<24								
HbA1c <5.7	3109	1035 (33.29%)	Reference			Reference		
5.7≤HbA1c<6.5	2615	916 (35.03%)	1.05(0.98-1.13)	0.08		1.09 (1.003- 1.18)	0.02	
HbA1c≥6.5	599	222 (37.06%)	1.11 (1.0-1.25)	0.03		1.18 (1.03-1.33)	0.01	
BMI≥24								
HbA1c <5.7	26	4 (15.38%)	Reference			Reference		
5.7≤HbA1c<6.5	36	6 (16.67%)	1.08(0.29- 6.79)	0.43		1.09 (1.0 - 1.18)	0.03	
HbA1c≥6.5	17	4 (23.53%)	1.53 (0.28-12.89)	0.27		1.18 (1.04-1.33)	0.006	
Vaginitis					0.23			0.22
0								
HbA1c <5.7	4473	968 (32.48%)	Reference			Reference		
5.7≤HbA1c<6.5	1606	861 (34.17%)	1.05 (0.98- 1.14)	0.09		1.08 (0.99-1.18)	0.04	
HbA1c≥6.5		202 (34.89%)	1.07 (0.95-1.22)	0.12		1.13(0.98-1.30)	0.047	
1								
HbA1c <5.7	155	71 (45.81%)	Reference			Reference		
5.7≤HbA1c<6.5	131	61 (46.56%)	1.02 (0.80-1.28)	0.43		0.98 (0.75-1.20)	0.46	
HbA1c≥6.5	37	24 (64.86%)	1.42 (1.03-1.85)	0.02		1.47 (1.07-1.91)	0.01	

Ajusted for age, BMI, abnormal pregnancy history, vaginitis and history of syphilis.

## Discussion

### Main results

Our large cross-sectional study analyzed the association between diabetes and prediabetes and HPV positivity, adjusting for confounders including age, BMI, history of adverse pregnancy, vaginitis, and history of syphilis. We found that both diabetic and prediabetes patients had different degrees of increased HPV positivity. Subgroup analyses showed a consistent association between diabetes and HPV infection in women with different characteristics, with a significant 47% higher risk of persistent HPV infection when diabetes was associated with vaginitis. At the same time, we found for the first time that there is a saturation effect on the effect of different blood glucose control levels on HPV persistent infection. Elevated HbA1c increases the risk of HPV persistent infection, but when HbA1c exceeds 5.7%, the effect is weakened. Our study suggests that high blood sugar levels may be one of the triggers of HPV persistent infection, and provides clinical epidemiological evidence for a deeper understanding of the mechanism of HPV persistent infection.

### Comparison with findings of previous studies

The results of existing studies are inconsistent, and there is no consensus on the relationship between diabetes and HPV infection. A study in Turkey found that diabetic patients (30-65 years old) had higher HPV 16 positivity than non-diabetic patients (10). A Danish study found that in patients with type 1 diabetes (15-49 years old), the incidence of genital warts caused by HPV infection was higher than that in non-diabetic patients (11), and diabetic patients with genital herpes had more severe infections, requiring long-term treatment (12). The prevalence of HPV infection was significantly higher in the diabetic group than in the non-diabetic group in southeastern Poland. HPV-DNA was detected in 19.1% of diabetic patients, compared with 8% in the control group. Viral infections were detected more frequently in patients with a history of type 2 diabetes for more than 10 years (13). A study using the U.S. Using the National Health and Nutrition Examination Survey (2015–2016) found no significant association between cervical HPV infection and diabetes after adjustment for age, sex, race, marital status, and the presence of comorbidities (14).

Our research is based on a cross-sectional study of cervical outpatient clinics in China’s largest academic woman’s hospital. Since we did not find an association between glycated hemoglobin and HPV infection in women aged 20-50, we only found an association between 20-50-year-old women. The association was found in women with cervical clinic visits. Among these patients over 50 years old, there were 3135 (48.97%) non-diabetic patients, 2651 (41.41%) prediabetes patients, and 616 (9.62%) diabetic patients. The HPV positive rate of diabetic patients was as high as 36.69%, and the HPV positive rate of non-diabetic patients was 33.14%.

Since the median age of onset of diabetes in China is 51.3 years (2), infection by HPV on the mucosal and skin surfaces of the host epithelium is usually self-limited (15), and there is a transient infection in young women, so we It is believed that HPV infection after the age of 50 can better represent the status of persistent HPV infection, and it is more clinically meaningful to study the relationship between diabetes mellitus and HPV infection after the age of 50. We believe that age-related self-limitation of HPV may explain the heterogeneity of the current findings. Hyperglycemia may be one of the triggers for persistent HPV infection.

### Interpretations

A picture emerges in which diabetes is not only a metabolic disease, but one with profound and sustained effects on immune cell function (16). Diabetic patients have altered T cell and macrophage proliferation, impaired NK cell and B cell function, and up-regulated macrophage polarization toward M1, manifesting as abnormal innate and adaptive immunity (17). The innate immune response plays a fundamental role in defending against invading pathogens through a myriad of humoral and cellular mechanisms; macrophages, epithelial cells, innate lymphoid cells, and neutrophils are key factors in initiating the adaptive immune response, impaired in diabetic patients The innate immune response increases susceptibility to pathogens (18).

A normal immune system clears most HPV infections quickly. However, hyperglycemia may contribute to virus-host interactions as well as changes in the immune system in diabetic patients, possibly increasing the risk of reactivation of latent HPV infections and/or reducing the ability to clear newly acquired HPV infections (19, 21–23).

Given the increased risk of various infections in people with diabetes, poor glycemic control appears to exacerbate this risk. Studies evaluating the effect of glycemic control (HbA1c) on HPV infection are therefore warranted, as good glycemic control has previously been shown to reduce the risk of other infections (24, 25). Combined with the above mechanistic studies, our study further provides clinical evidence that diabetes is a potential cause of persistent HPV infection.

### Strength and limitations

Our study was a large cross-sectional study using relatively recent data (2019–2022) reflecting current disease patterns and trends; adjusted for age, BMI, adverse maternal history, vaginitis, syphilis History-inclusive risk factors reduce selection bias. Our data provide clinicians with additional evidence that women over 50 years of age with diabetes are at higher risk for persistent HPV infection. Using single and stable glycated hemoglobin to assess glycemic status is more clinically attractive.

Our study also has some limitations. First, the cross-sectional data cannot be used to determine causal relationships. Second, retrospective studies are imperfect and lack information on lifestyle-related factors such as sexual behavior and smoking. But we included a history of syphilis to make up for the lack of an adverse sexual life history, and the smoking rate of Chinese women is about 2.1%, which is at a very low level (26). These unmeasured confounds such as parity, the status of their HPV vaccination, other risky behaviors may have caused us to overestimate the RR between diabetes and HPV. But we used E-value sensitivity analysis to quantify the potential effect of unmeasured confounders (20) and found that unmeasured confounders were unlikely to explain the overall effect.(E value=1.64). Next, our study is a single-center study with a population of cervical outpatients over 50 years old in China and more multicenter studies are needed to confirm in the future. Finally, with diabetes, we do not differentiate between type 1 and type 2.

## Conclusion

The findings of this study have important clinical and public health implications. Our study found a higher risk of persistent HPV infection among women with diabetes over the age of 50. There are differences in the HPV positive rate among people with different blood sugar levels. This difference suggests that clinicians need to pay attention to the blood sugar control of HPV positive patients. For women over 50 years old with hyperglycemia, cervical cancer screening strategies need to be re-established.

## Data availability statement

The raw data supporting the conclusions of this article will be made available by the authors, without undue reservation.

## Ethics statement

The studies involving human participants were reviewed and approved by Ethics Committee of the Obstetrics & Gynecology Hospital of Fudan University (2022-78). The data are anonymous, and the requirement for informed consent was therefore waived.

## Author contributions

CYY analyzed the data, drafted the manuscript and contributed to study design. CYZ contributed to data collation. CMY and HJ revised the article. All authors contributed to the article and approved the submitted version.

## Funding

This work was supported by the program for National Natural Science Foundation of China (No. 81902131) and Shanghai “Rising Stars of Medical Talents” Youth Development Program (SHWRS (2020)_087).

## Conflict of interest

The authors declare that the research was conducted in the absence of any commercial or financial relationships that could be construed as a potential conflict of interest.

## Publisher’s note

All claims expressed in this article are solely those of the authors and do not necessarily represent those of their affiliated organizations, or those of the publisher, the editors and the reviewers. Any product that may be evaluated in this article, or claim that may be made by its manufacturer, is not guaranteed or endorsed by the publisher.
